# CD60b: Enriching Neural Stem/Progenitor Cells from Rat Development into Adulthood

**DOI:** 10.1155/2017/5759490

**Published:** 2017-11-15

**Authors:** Fernanda Gubert, Camila Zaverucha-do-Valle, Michelle Furtado, Pedro M. Pimentel-Coelho, Nicoli Mortari, Ana C. M. Leão, Elize A. Hayashi, Alberto Nobrega, Rosalia Mendez-Otero, Marcelo F. Santiago

**Affiliations:** ^1^Instituto de Biofísica Carlos Chagas Filho, Universidade Federal do Rio de Janeiro, 21941-902 Rio de Janeiro, RJ, Brazil; ^2^Departamento de Imunologia, Instituto de Microbiologia Paulo de Góes, Universidade Federal do Rio de Janeiro, 21941-902 Rio de Janeiro, RJ, Brazil

## Abstract

CD60b antigens are highly expressed during development in the rat nervous system, while in the adult their expression is restricted to a few regions, including the subventricular zone (SVZ) around the lateral ventricles—a neurogenic niche in the adult brain. For this reason, we investigated whether the expression of C60b is associated with neural stem/progenitor cells in the SVZ, from development into adulthood. We performed *in vitro* and *in vivo* analyses of CD60b expression at different stages and identified the presence of these antigens in neural stem/progenitor cells. We also observed that CD60b could be used to purify and enrich a population of neurosphere-forming cells from the developing and adult brain. We showed that CD60b antigens (mainly corresponding to ganglioside 9-O-acetyl GD3, a well-known molecule expressed during central nervous system development and mainly associated with neuronal migration) are also present in less mature cells and could be used to identify and isolate neural stem/progenitor cells during development and in the adult brain. A better understanding of molecules associated with neurogenesis may contribute not only to improve the knowledge about the physiology of the mammalian central nervous system, but also to find new treatments for regenerating tissue after disease or brain injury.

## 1. Introduction

As yet, no efficient treatment exists for most lesions or degenerative diseases of the central nervous system (CNS). The discovery that neurogenesis persists in the adult mammalian CNS was a breakthrough in medical science, with several potential therapeutic implications, such as the possibility of regenerating the CNS with endogenous newly generated neurons [1–4]. Since then, many researchers have been attempting to develop strategies to stimulate the generation of new neurons in different pathological conditions [3, 5].

The subventricular zone (SVZ) is one of the few neurogenic regions that persist in the adult mammalian brain [3, 6]. This region appears during embryonic development, just above the ventricular zone (VZ), and remains throughout adulthood, although it becomes thinner after several cytoarchitectural changes that occur during the perinatal period [6, 7]. Three main cell populations have been described in the adult SVZ: B cells, which are slowly proliferating neural stem cells that originate rapidly proliferating transit-amplifying C cells, which in turn give rise to A cells, neuroblasts that leave the SVZ and migrate to different sites, such as the olfactory bulb [8].

Neural stem cells are usually identified *in vitro* by their ability to generate neurospheres in the presence of growth factors [9]. Although the unique biology of these cells has been studied by many groups, several questions remain, such as the identity of the bona fide neural stem cells. This difficulty results from the existence of different neural progenitors in various stages of maturation in the neurogenic zones [3].

Gangliosides are glycosphingolipids that contain sialic acid; they are present in the cytoplasmic membrane and are highly abundant in the CNS [10]. Gangliosides are an important class of molecules that control many steps in the formation of the complex adult brain structure, including proliferation, migration, neuritogenesis, axonal outgrowth, and synaptic transmission [11].

The ganglioside 9-O-acetyl GD3 (9acGD3), which is recognized by anti-CD60b antibodies, is formed from the acetylation of the ganglioside GD3 by the action of the enzyme encoded by *CASD1* gene, recently identified as essential for sialic acid 9-O-acetylation [12, 13]. CD60b are expressed in the developing brain, in a pattern that correlates spatiotemporally with events of cell migration and/or axonal extension, in the retina, superior colliculus, hippocampus, cerebellum, and telencephalon [14–17]. Functionally, it has been shown that blockage of CD60b with a specific antibody halts the advance of growth cones from dorsal root ganglia neurons [18]. Immunoblockage of CD60b also inhibits the radial migration of cerebellar granule cells in the developing rat cerebellum [16, 19] and tangential neuroblast migration from postnatal SVZ explants [20]. Furthermore, CD60b-dependent calcium signaling through purinergic receptors was described as crucial for the migration of granular cell precursors during development [21]. In the adult peripheral nervous system, CD60b is re-expressed in the sciatic nerve after a crush injury, and its expression correlates with the axonal outgrowth through the lesion site [22].

In the rat embryonic telencephalon, CD60b is highly expressed around the ventricles and in radially oriented processes [14, 23], and this expression decreases during the first postnatal week. In adult rodents, CD60b antigens are no longer expressed in most regions of the CNS, persisting in the SVZ, rostral migratory stream, retina, and cerebellum [15, 24].

It is known that CD60b is expressed in embryonic neuroepithelial cells [25] and neurospheres generated from embryonic stem cell-derived neural stem cells [26]. Considering that adult neurogenesis shares several characteristics with embryonic and early postnatal events, in this study we investigated whether CD60b was expressed by neural stem cells in the adult SVZ. We also investigated whether CD60b could be used to isolate a cell population enriched in neurosphere-forming neural stem/progenitor cells, from embryonic development to adulthood.

## 2. Experimental Procedures

### 2.1. Animals

All experimental procedures were carried out in accordance with the guidelines of the Federal University of Rio de Janeiro and were approved by the Animal Care Committee of this Institution. All efforts were made to reduce the number of animals used and their suffering. Embryonic and postnatal Lister Hooded rats of different ages (embryonic day (E) 14.5, E16, postnatal day (P) 0, P7, P21, and 3-month-old young adults) were used in this study.

### 2.2. BrdU Labeling

For BrdU experiments, adult rats received intraperitoneal BrdU injections (50 mg/kg) every 2 h for 16 h and were euthanized 2 h after the last injection.

### 2.3. Neurosphere Assay

For the E16 neurosphere assay, the telencephalon was dissected and mechanically dissociated with a Pasteur pipette in Dulbecco's Modified Eagle's Medium/Nutrient Mixture F-12 (DMEM-F12; Thermo Fisher, Waltham, MA, USA). For the P21 and adult neurosphere assays, SVZ was microdissected, and then incubated, with a dissociation solution containing 294.6 mg/ml papain (Worthington, Lakewood, NJ, USA), 10% bovine serum albumin (BSA) (Worthington), 160 mg/ml DNAse (Worthington), and 150 mg/ml L-cystein (Worthington) in 25 ml of DMEN-F12 for 30 min in 5% CO_2_/95% air at 37°C. After incubation, the cells were mechanically dissociated and counted with a Neubauer chamber.

After dissociation, the cells were centrifuged at 300 ×g and then incubated on ice for 30 min with the following primary antibody: mouse IgM anti-CD60b (5 *μ*l per 10^6^ cells; Santa Cruz Biotech, Dallas, Texas, USA) diluted in phosphate-buffered saline (PBS). After incubation, cells were washed 2x with PBS and centrifuged at 300 ×g, followed by incubation for 30 min with the secondary antibody: mouse anti-IgM conjugated with superparamagnetic beads (20 *μ*l per 10^7^ cells; Miltenyi Biotec, Bergisch Gladbach, Germany) diluted with PBS containing 10% BSA.

After incubation, cells were washed 2x with PBS, suspended in neurosphere medium, and loaded onto MACS separation columns (Miltenyi Biotec). Antibody-labeled cells were retained in the column under the magnetic field, and nonlabeled cells that did not attach in the column were saved. After the column was removed from the magnetic field, positive CD60b cells were obtained by washing the column with fresh neurosphere medium. The neurosphere medium composition was DMEM-F12 supplemented with B27 Supplement Minus Vitamin A (Invitrogen, Carlsbad, CA, USA), 10 ng/ml basic FGF (Invitrogen), 20 ng/ml EGF (Invitrogen), 30% glucose, 2 mM glutamine (Invitrogen), and 1% penicillin and streptomycin (Invitrogen).

Cells were plated in a 24-well plate (10 cells/*μ*l–400 *μ*l) for 7 days in 5% CO_2_/95% air at 37°C. During this 7-day period, the plates were not moved, and after this period, neurospheres were photographed in an EVOS® microscope (Thermo Fisher) for quantification. Statistical analysis was performed using the unpaired two-tailed *t*-test in GraphPad Prism version 4.02 for Windows (GraphPad Software Inc.). Statistical significance was considered when *p* ≤ 0.05. All data are presented as mean ± standard error of the mean (SEM).

For secondary and tertiary neurosphere formation, primary and secondary neurospheres were dissociated with papain, as described previously. The number of cells was counted in a Neubauer chamber, and dissociated cells were plated in a 24-well plate (10 cells/*μ*l–400 *μ*l) for 7 days in 5% CO_2_/95% air at 37°C. During this 7-day period, the plates were not moved, and after this period, they were analyzed.

For differentiation assay, neurospheres (E16 and adult) derived from both negative and positive populations were mechanically dissociated to a single-cell suspension and replated (2 × 10^4^ cells/well) on coverslips previously treated with poly-L-lysine (PLL, 100 *μ*g/ml; Sigma) and laminin (20 *μ*g/ml; Invitrogen). They were maintained in Neurobasal medium, without growth factors, supplemented with B27 (Invitrogen), glutamine (2 mM), N2 (Invitrogen), and penicillin and streptomycin (40 mg/ml) for 5 days in 5% CO_2_/95% air at 37°C. Later, the cells were washed and fixed with 4% *p*-formaldehyde (PFA) at room temperature for immunocytochemistry analysis.

### 2.4. Flow Cytometry Analysis

Analyses were performed on a FACS CALIBUR dual-laser flow cytometer (Becton-Dickinson, San José, CA, USA). Gating parameters were set by side and forward scatter to eliminate debris and dead and aggregated cells. The SVZ was dissected and dissociated with papain as described for the neurosphere essay. Cells were suspended in a 1 : 100 dilution of mouse monoclonal IgM antibody against 9-O-acetyl GD3 for 20–25 min on ice, washed, and incubated in PE-conjugated goat anti-mouse secondary antibody (Jackson, West Grove, PA, USA).

### 2.5. Immunohistochemistry

Pregnant Lister Hooded rats were anesthetized and euthanized. Briefly, E14.5 embryos were removed and their brains were dissected and fixed in 4% PFA overnight and then transferred to a cryoprotective solution (30% sucrose in PBS). P0, P7, P21, and adult animals were anesthetized and fixed by cardiac perfusion with a solution of 4% PFA, and their brains were removed and transferred to the cryoprotective solution. Coronal sections (14–20 *μ*m thick) were cut on a cryostat (Leica Microsystems) and mounted on gelatin-covered slides. Sections were stored at −20°C until immunohistochemistry processing.

For immunostaining, brain sections were washed three times with 0.01% Triton X-100 in PBS and incubated with a blocking solution (5% normal goat serum in PBS) for 30 min at room temperature. The sections were incubated with the primary antibody mouse IgM anti-CD60b (1 : 100, from Santa Cruz Biotechnology or Jones from Sigma, St. Louis, MO, USA) alone or in combination with one of the following primary antibodies or lectin: anti-nestin (1 : 100, Chemicon, Temecula, CA, USA), anti-vimentin (1 : 200, Chemicon), anti-GFAP (1 : 500, Dako, Denmark), anti-EGFR (1 : 250, Abcam, Cambridge, MA, USA), anti-doublecortin (1 : 500, Chemicon), PNA (Peanut agglutinin; 1 : 100, Sigma) overnight at 4°C.

After washes with 0.01% Triton X-100 in PBS, sections were incubated with the secondary antibody goat anti-mouse IgM Cy3-conjugated (1 : 500, Jackson) alone or in combination with one of the following secondary antibodies: goat anti-rabbit Alexa Fluor 488-conjugated (1 : 500, Invitrogen) or goat anti-mouse IgG Alexa Fluor 488-conjugated (1 : 500, Invitrogen), for 2 h at room temperature. Nuclei were counterstained with TO-PRO-3 (1 : 1000; Thermo Fisher). After washing with PBS, slides were mounted with VectaShield (Vector, Burlingame, CA, USA) and analyzed and photographed on a confocal microscope (Zeiss LSM 510 Meta, Zeiss).

### 2.6. Immunocytochemistry

For immunocytochemistry of SVZ cells, adult brains were removed, SVZ was dissected, and cells were dissociated and separated through MACS as described in the “neurosphere assay” item. 9-acGD3+ cells were centrifuged in 5 × 10^5^ cells/ml concentration for 3 minutes at 600 rpm and transferred to gelatin-covered slides. Cells were fixed with 4% PFA for 15 minutes and kept at −20°C for further analysis.

Immunocytochemistry was performed both in SVZ cells and differentiated cells derived from neurospheres. The protocol was similar to the one used for immunohistochemistry and the following primary antibodies were used: mouse IgM anti-CD60b (1 : 100, Santa Cruz), anti-GFAP (1 : 500, Dako, Denmark), anti-doublecortin (1 : 500, Chemicon), anti-nestin (1 : 100, Chemicon, Temecula, CA, USA), anti-NG2 (1 : 500, Millipore), Tuj1 (1 : 500, Covance), and anti-BrdU (1 : 20, Amersham, EUA).

### 2.7. Neurosphere Staining

Cells and neurospheres were fixed with 4% PFA for 30 min at room temperature. After washing in PBS, samples were incubated with 5% normal goat serum 0.01% Triton X-100 in PBS for 30 min at room temperature, with the following primary antibodies: mouse IgM anti-CD60b (1 : 100; from Santa Cruz Biotech, or Jones from Sigma); mouse IgG anti nestin (1 : 100; Abcam); rabbit IgG anti-GFAP (1 : 300, Dako); mouse IgG anti-*β*-tubulin Isotype III (Tuj1; 1 : 100; Covance, Princeton, NJ, USA); rabbit IgG anti-NG2 (1 : 500; Millipore). After three washes with PBS, they were incubated for 2 h at room temperature with the secondary antibodies: Cy3 conjugated goat anti-mouse IgG or IgM and Alexa 488-conjugated goat anti-rabbit. Nuclei were counterstained with TO-PRO-3. After washing with PBS, slides were mounted with VectaShield (Vector) and analyzed and photographed with the aid of a confocal microscope (Zeiss LSM 510 Meta, Zeiss) or an Axiovert 135 epifluorescence microscope equipped with an HRc Axiocam (Zeiss).

## 3. Results

### 3.1. CD60b Expression Decreases throughout Cortical Development

**Figure 1:**
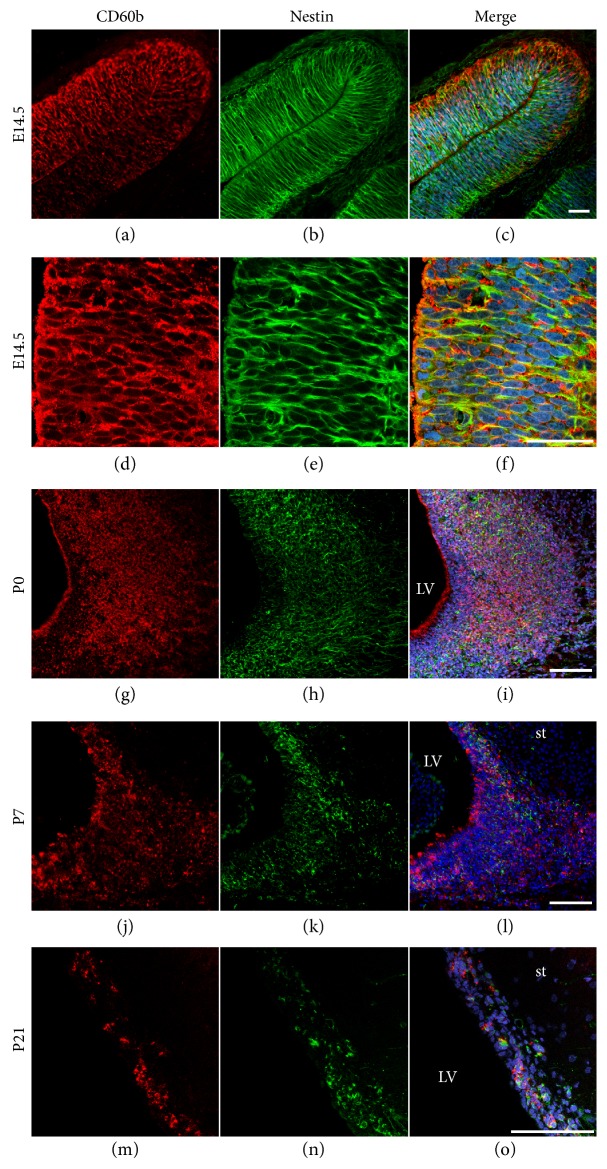
CD60b expression decreases throughout cortical development. Confocal photomicrographs showing the expression of 9-O-acetyl GD3 (CD60b in red) and nestin (in green) in the developing cerebral cortex of E14.5 (a–f), P0 (g–i), P7 (j–l), and P21 (m–o) rats. In E14.5, CD60b is expressed in cell bodies and radially oriented structures (a–c) and its distribution is very similar to nestin distribution (d–f). From P0 to P21, there is a progressive decrease in CD60b expression, but its distribution is still similar to nestin distribution. Nuclei were stained with TO-PRO-3 (blue). Scale bars: 50 *μ*m (a–f) and 100 *μ*m (g–o). st: striatum; LV: lateral ventricle.

The expression of CD60b antigens was evaluated by immunohistochemistry in coronal and parasagittal sections of the developing rat telencephalon at E14.5, P0, P7 and P21. At E14.5, we observed the expression of CD60b in cell bodies and in radially oriented structures throughout all compartments of the developing cerebral cortex, including the VZ (Figures 1(a), 1(b), and 1(c)). At this stage, the distribution of CD60b was very similar to the distribution of nestin, a neural stem/progenitor cell marker (Figures 1(d), 1(e), and 1(f)). However, the pattern of labeling was different, with CD60b showing a punctiform pattern, whereas nestin showed a more continuous staining pattern. This is due to the fact that 9acGD3 is probably located in lipid rafts of the cell membrane, while nestin is a component of the cellular cytoskeleton. From P0 to P21, we observed a progressive decrease in the expression of CD60b in the developing cerebral cortex (Figures 1(g), 1(j), and 1(m)). After the first postnatal week, CD60b expression was mostly confined to the SVZ and could not be observed in the cortical layers (Figures 1(j) and 1(m)). This distribution of CD60b correlated with the location of nestin-positive cells (Figures 1(i), 1(l), and 1(o)).

**Figure 2:**
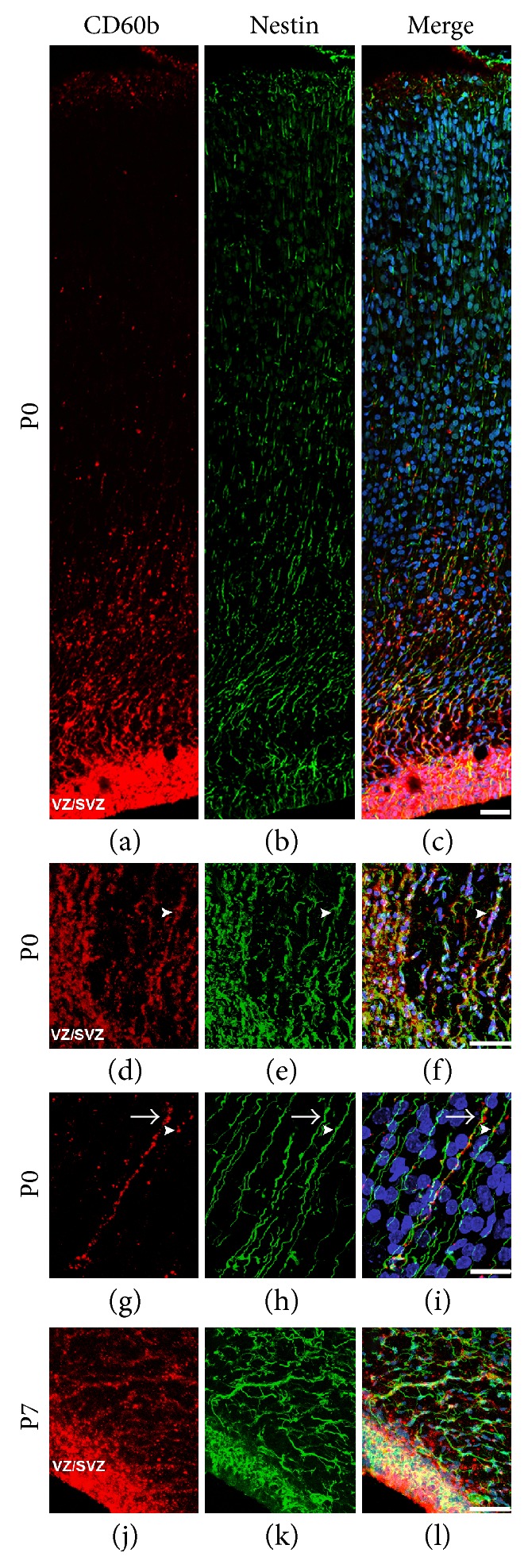
CD60b expression in radial glial cells in the newborn brain. Confocal images of early postnatal forebrain cryosections showing the expression of CD60b (in red) and of the intermediate filament nestin (in green) in the developing cerebral cortex of P0 (a–i) and P7 (j–l) rats. The photomontage in (a–c) shows the pattern of expression of CD60b in the P0 brain. Note that the ganglioside is expressed in nestin-positive cells in the VZ/SVZ and in the proximal part of radial glial cell processes, as shown in high magnification images of P0 (d–f) and P7 brains (arrowheads (j–l)). Arrowhead in (d–f) shows an example of a radial glial cell process expressing both nestin and CD60b. In the occipital cerebral cortex, CD60b is still expressed in the distal part of a few radial glial cell processes (g–i) with 2 distinct patterns: while several processes have a punctacte expression of the ganglioside (arrowheads in (g–i)), we also observed a strong and continuous expression along other few processes (arrow in (g–i)). Scale bar: 50 *μ*m (a–f, j–l) or 20 *μ*m (g–i). Nuclear staining with TO-PRO-3 in blue (c, f, i, and l). VZ/SVZ: ventricular zone/subventricular zone.

In a detailed analysis, we studied the distribution of CD60b along the radial glial cell processes, the main progenitor cell during nervous system development. We observed that, at P0, this ganglioside is mainly expressed at the proximal (close to the lateral ventricle) part of these processes, but rarely present in the distal (close to the pial surface) parts (Figures 2(a), 2(b), and 2(c)). In addition, we found several cells in close association with radial glial cell processes coexpressing nestin and CD60b leaving the SVZ at P0 (arrowhead in Figures 2(d), 2(e), and 2(f)), suggesting that CD60b is expressed in radial glial cell processes that are supporting cell migration in the newborn brain. At P0, while radial glial cell distal processes were still nestin positive in the cerebral cortex, only a few of these processes were CD60b positive. An example of a few radial glial cell processes expressing both antigens in the cerebral cortex is showed in a higher magnification in Figures 2(g), 2(h), and 2(i), where one of the processes is strongly positive for the ganglioside (arrow), while others have a punctate expression (arrowhead). At P7, some nestin-positive processes still express CD60b at SVZ (arrowheads) (Figures 2(j), 2(k), and 2(l)).

### 3.2. CD60b Is Expressed in Proliferative Progenitor Cells in the Adult SVZ

**Figure 3:**
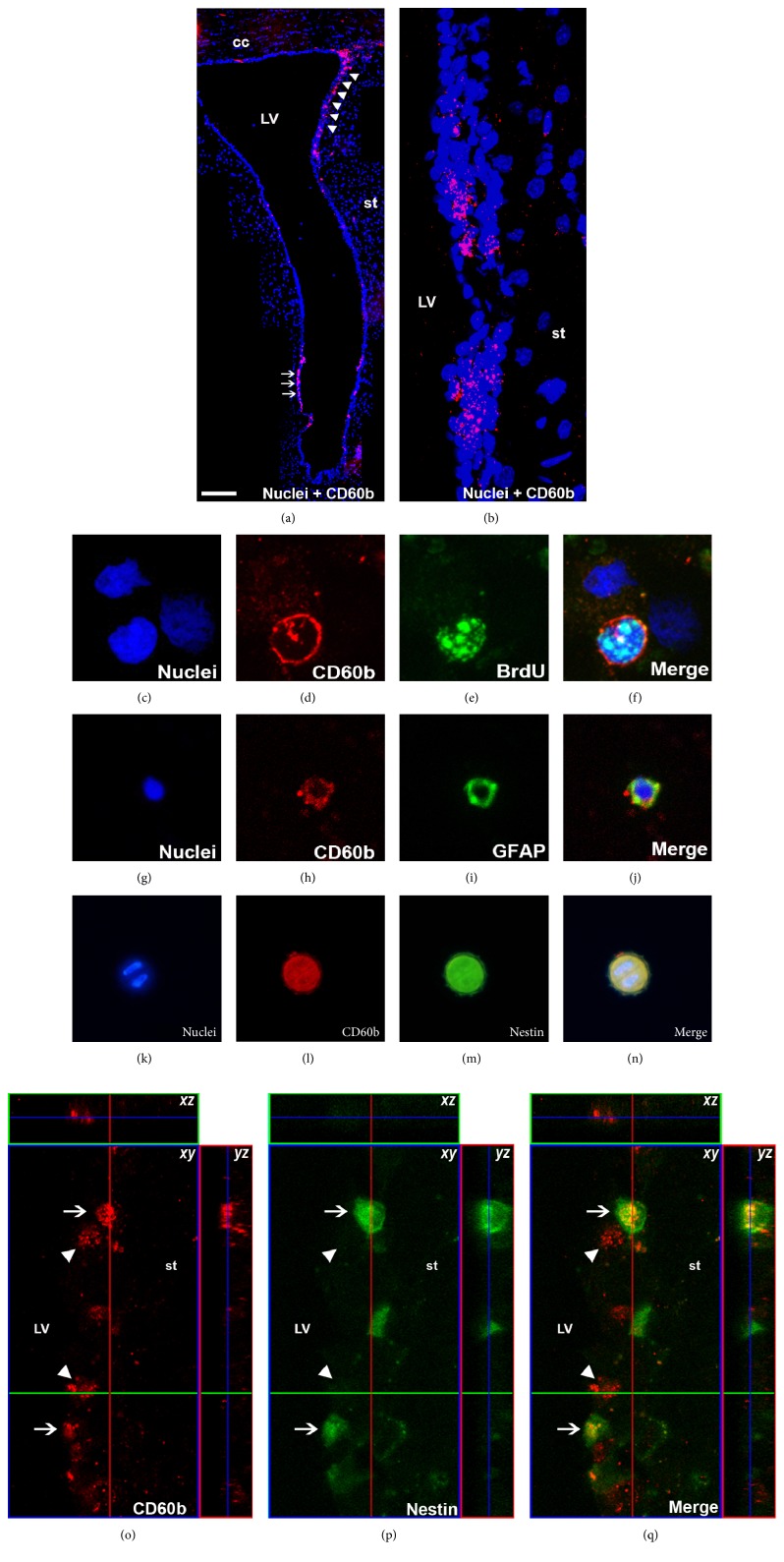
CD60b is expressed along rat adult subventricular zone in proliferative and stem/progenitor cells. (a) Confocal image of a coronal section of the lateral ventricle of adult rat showing expression of CD60b in red. We can note cells expressing CD60b antigens in the lateral wall near the RMS (arrowheads) and cells CD60b-positive in the medial wall of the lateral ventricle (arrows). (b) High magnification of the SVZ showing punctiform expression of CD60b in SVZ cells along the lateral wall. We can notice that not all the cells express CD60b. (c–n) Confocal images of cells dissociated from the subventricular zone of adult rat stained with TO-PRO-3 (in blue; c, g, and k), showing the expression of CD60b (in red; (d, h, and l)) costained for BrdU (e), GFAP (i), or nestin (m) in green. Merge images are show in (f, j, and n). (o–q) Confocal Z-stack reconstruction and orthogonal slices (superior and lateral boxes) showing cells double-labeled (arrows in q) for CD60b in red (o) and nestin in green (p). Merge images showing colocalization in (q). We can also note CD60b-positive cells that do not express nestin (arrowheads in (q)). Scale bar: 300 *μ*m (a); 30 *μ*m (b); 12 *μ*m (c–q). cc: corpus callosum; LV: lateral ventricle; st: striatum.

Immunostaining of coronal sections of adult rat brains showed expression of CD60b around the lateral ventricles, mainly in the dorsalateral SVZ (arrowheads in Figure 3(a)). The staining revealed a subpopulation of cells, distributed over the lateral walls of the lateral ventricle, which reflects the lower expression of CD60b in the adult SVZ. In the medial wall, it is possible to note a discrete subpopulation of CD60b-positive cells (Figure 3(a), arrows). In a higher magnification, the punctiform CD60b expression can be visualized in the SVZ of the lateral wall of the lateral ventricle (Figure 3(b)).

To quantify the number of CD60b-positive cells in the adult SVZ, the area around the lateral ventricle was microdissected and cells were dissociated, stained with an anti-CD60b antibody, and analyzed by flow cytometry. We observed that 0.69 ± 0.29% (*n* = 4) of cells from the lateral wall of the lateral ventricle were positive for this ganglioside.

Likewise in the developmental period, CD60b was expressed in nestin-positive cells in adult SVZ (Figures 3(k), 3(l), 3(m), 3(n), 3(o), 3(p), and 3(q), arrows), suggesting that it continues to be expressed by neural stem/progenitor cells. However, we also noted CD60b-positive cells that were negative for nestin (Figures 3(o), 3(p), and 3(q), arrowheads).

**Figure 4:**
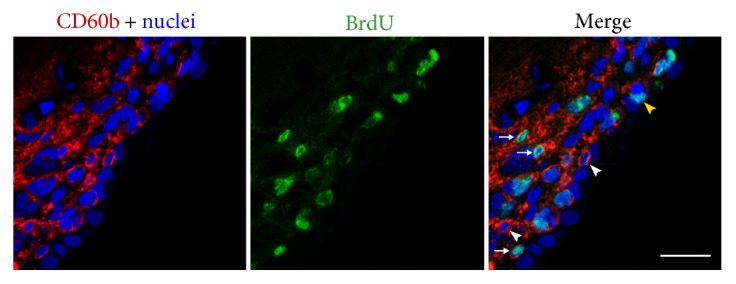
CD60b is expressed in proliferating cells in the rat adult subventricular zone. Confocal image of a coronal section of the lateral ventricle of an adult rat that received intraperitoneal BrdU injections (50 mg/kg) every 2 h for 16 h and was euthanized 2 h after the last injection. We can observe cells expressing only CD60b (red; arrowheads), cells expressing only BrdU (green, yellow arrowhead), and cells double-labeled for CD60b and BrdU (arrow). Nuclei are counterstained with TO-PRO-3 in blue. Scale bar: 20 *μ*m.

In order to confirm that neural progenitor cells express CD60b, adult rats were intraperitoneally injected with BrdU (50 mg/kg) every 2 h for 16 h. Shortly after the last injection, the animals were euthanized, SVZ was microdissected and dissociated, and the cells were fixed with PFA and stained with an antibody against BrdU and CD60b. We observed double-positive cells (Figures 3(c), 3(d), 3(e), and 3(f)), indicating that CD60b is expressed in proliferating cells. We also could observe, using cytospin-isolated SVZ cells, colocalization of CD60b and GFAP (Figures 3(g), 3(h), 3(i), and 3(j)) and CD60b and nestin (Figures 3(k), 3(l), 3(m), and 3(n)). GFAP, an intermediate filament expressed by astrocytes, also stains B cells in the SVZ, and nestin is expressed by neural stem/progenitor cells. Proliferating CD60b-positive cells were also observed by immunohistochemistry. In Figure 4, we can notice double-positive cells for BrdU and CD60b (arrows), but we can also see single-labeled cells, that were only positive for CD60b (white arrowheads) or BrdU (yellow arrowhead), indicating that not all proliferating cells express CD60b.

### 3.3. CD60b Is Expressed in Different Populations in the Adult SVZ

**Figure 5:**
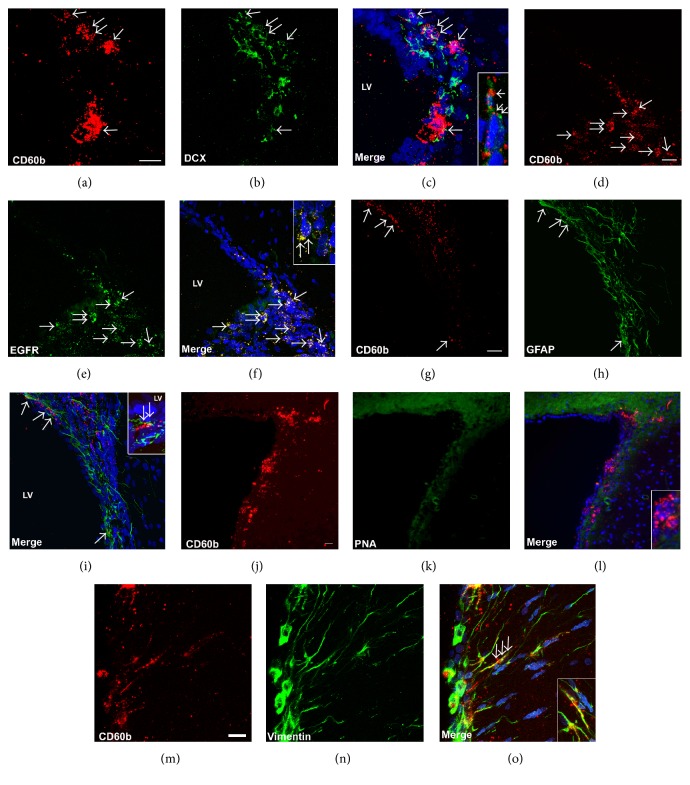
CD60b is expressed in different populations in the adult SVZ. Confocal images of adult SVZ sections showing the expression of CD60b (a, d, g, j, and m in red) and doublecortin (b-c), EGFR (e-f), GFAP (h-i), PNA (k-l), and vimentin (n-o) (in green). Nuclei were counterstained with TO-PRO-3 (blue). We observed the expression of CD60b in DCX (expressed in neuroblasts), R-EGF (expressed mostly in type C cells), GFAP (expressed in type B cells and astrocytes), and vimentin-positive cells (expressed in radial glia-like cells) (arrows). PNA, which was described as a negative marker of neural stem cells, and CD60b showed little colocalization (l). High magnification is showed in the insets. LV: lateral ventricle; DCX: doublecortin. Scale bar: 20 *μ*m.

**Figure 6:**
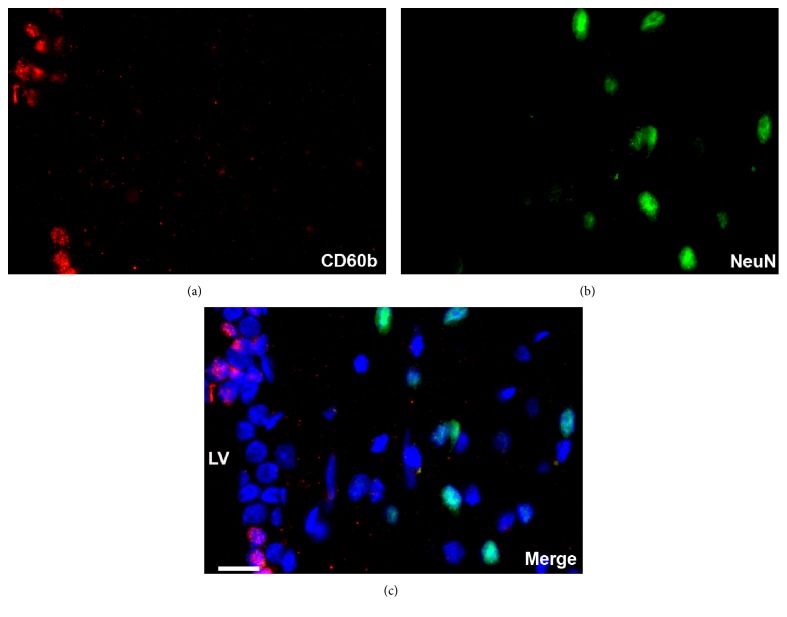
CD60b did not stain mature neurons. Confocal images of adult SVZ section showing the expression of CD60b ((a) in red) and NeuN ((b) in green). Nuclei stained with TO-PRO-3 in blue. Merge images showed no costained was observed between the two markers, demonstrating that CD60b is not expressed in mature neurons (c). CD60b expression could be observed only in the SVZ, lining the lateral ventricle. LV: lateral ventricle. Scale bar: 20 *μ*m.

To further identify which type of neural progenitor cells express CD60b in the adult SVZ, we double-stained brain sections with antibodies against established markers of the three main cell types found in the SVZ. While doublecortin is expressed in neuroblasts (type A cells), EGFR is expressed mostly in C cells, and also in B cells, and GFAP is expressed in B cells. We noted the expression of CD60b in cells that were also positive for any of these three markers (Figure 5), showing that this ganglioside is not expressed in a particular cell type. However, we observed a major colocalization between CD60b and EGFR (Figures 5(d), 5(e), and 5(f), arrows). We also performed a double-staining analysis using markers of mature neurons, but we did not detect any colocalization (Figure 6), supporting the hypothesis that CD60b is expressed only in immature cells.

At this moment, there is no exclusive positive marker for B cells, the neural stem cells in the adult SVZ. As we mentioned before, B cells express GFAP; however, mature astrocyte also express this antigen. PNA, described as a negative marker for neural stem cells [27], showed very little overlap with CD60b (Figures 5(j), 5(k), and 5(l)), suggesting again that the CD60b stains neural stem cells. B cells, with stem cell capacity, have a radial morphology and are originated from radial glia [28]. To verify if CD60b is expressed in cells with radial glia-like phenotype, we double-stained the tissue with anti-CD60b and anti-vimentin antibodies. Vimentin is an intermediate filament expressed during development in radial glial cells and also in radial glia-like cells in adulthood [29]. We observed CD60b expression in adult radial glia-like cells, including their processes (Figures 5(m), 5(n), and 5(o)). These data also suggest that CD60b is expressed by B cells/neural stem cells.

### 3.4. CD60b Can Be Used to Obtain an Enriched Population of Progenitor Cells

**Figure 7:**
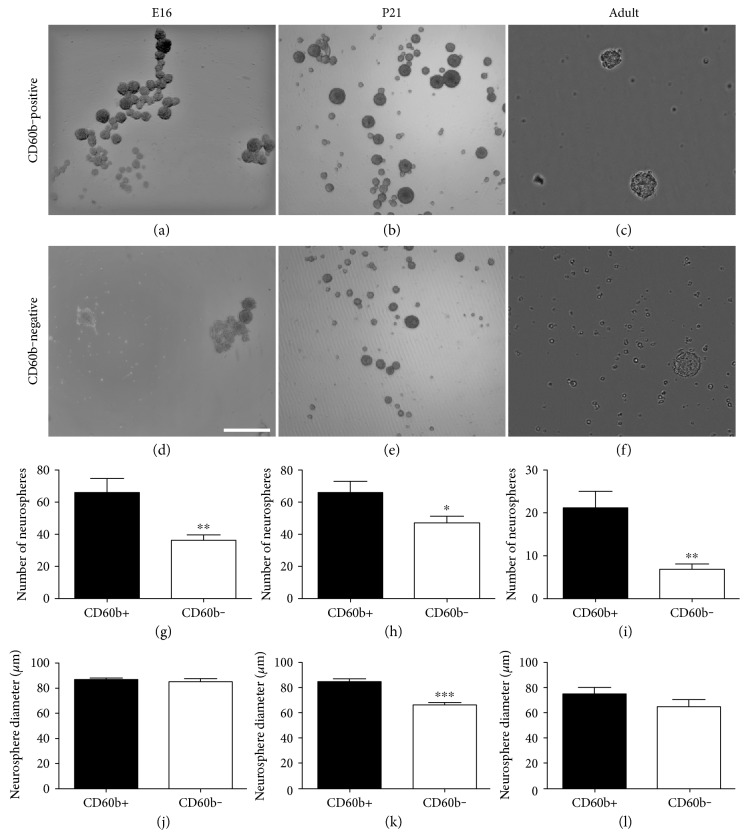
Quantification of neurospheres derived from CD60b-positive and CD60b-negative cells. (a–c) Neurospheres derived from CD60b-positive cells from E16 (a), P21 (b), and adult (c) rats. (d–f) Neurospheres derived from CD60b-negative cells in E16 (d), P21 (e), and adult (f) rats. (g–i) Quantification of the number of neurospheres derived from CD60b-positive and CD60b-negative cells in E16 (g), P21 (h), and adult (i) rats. We can note that CD60b-positive cells were able to generate more neurospheres when compared to the negative population in all the tested developmental stages. (j–l) Quantification of the diameter of neurospheres derived from CD60b-positive and CD60b-negative cells in E16 (j), P21 (k), and adult (l) rats. We can note that in P21 animals, the diameter of neurospheres generated from CD60b-positive cells was higher than the diameter of neurospheres generated from CD60b-negative cells. Scale bar: 1000 *μ*m (a-b and d-e); 200 *μ*m (c, f). ^∗^*p* < 0.05; ^∗∗^*p* < 0.01; ^∗∗∗^*p* < 0.001.

SVZ neural stem cells can be identified *in vitro* by their ability to generate neurospheres [9, 30]. Next, we examined whether the subpopulation of CD60b-positive cells included neurosphere-generating cells. SVZ cells were purified by MACS and cultured under neurosphere-promoting conditions. The number and diameter of the neurospheres generated from CD60b-positive and CD60b-negative cells isolated from embryonic VZ (E16), postnatal (P21), and adult SVZ were quantified. In E16, we observed significantly (*p* < 0.01) more neurospheres obtained from CD60b-positive cells (65.94 ± 8.78 cells/well, *n* = 3) than from negative ones (36.26 ± 3.32 cells/well, *n* = 3) (Figure 6). However, there was no difference in the diameter of the neurospheres obtained from CD60b-positive cells (86.67 ± 5.78 *μ*m, *n* = 3) compared to CD60b-negative cells (84.98 ± 10.59 *μ*m, *n* = 3) (Figure 6). On the contrary, in the P21 stage we noted that the neurospheres from CD60b-positive cells had significantly (*p* < 0.001) larger diameters (84.37 ± 2.45 *μ*m, *n* = 3) compared with those from CD60b-negative cells (66.37 ± 1.63 *μ*m, *n* = 3) (Figure 7). In this stage, we also observed that the number of neurospheres generated from CD60b-positive cells was significantly (*p* < 0.05) larger (66.06 ± 6.85, *n* = 3) than the number of neurospheres from CD60b-negative cells (47.15 ± 4.10, *n* = 3) (Figure 7). Moreover, in adult rats, the number of neurospheres in CD60b-positive cells was significantly larger (21.20 ± 3.84, *n* = 3) than in CD60b-negative cells (6.85 ± 1.15, *n* = 3), although there was no significant difference in the diameter of the neurospheres from CD60b-positive (75.60 ± 5.23 *μ*m, *n* = 3) and CD60b-negative (65.36 ± 5.78 *μ*m, *n* = 3) cells at this stage (Figure 7).

**Figure 8:**
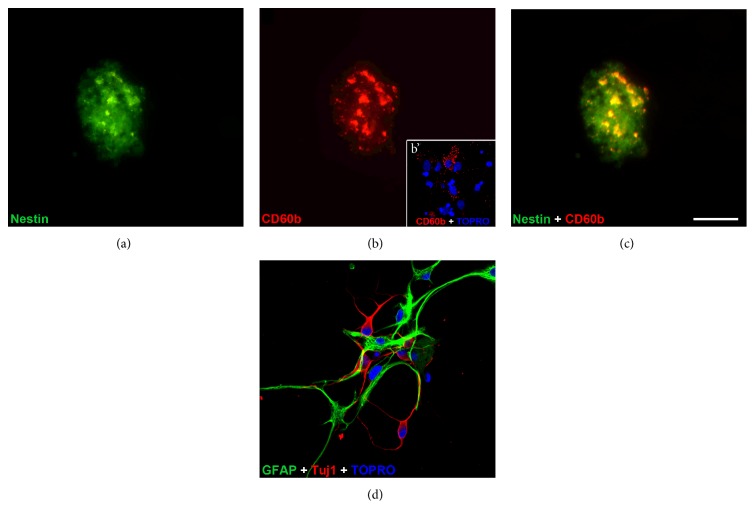
Characterization and differentiation of neurospheres derived from CD60b-positive cells. Immunostaining of neurospheres derived from CD60b-positive cells (a–c). As expected, most of the cells inside the neurosphere express nestin (green); a subpopulation of these cells also expresses CD60b (red). Some cells differentiated from neurospheres also expressed CD60b (b'). (d) Differentiation of neurospheres originated from CD60b-positive adult cells in GFAP-positive astrocytes (green) and TUJ1-positive neurons (red). Nuclei are stained with TO-PRO-3 in blue. Scale bar: 40 *μ*m (a–c); 46 *μ*m (b'); 35 *μ*m (d).

We also performed immunohistochemistry tests for CD60b and nestin in the neurospheres obtained from the adult SVZ (Figures 8(a), 8(b), and 8(c)). We observed that most of the CD60b-positive cells in the neurospheres were also positive for nestin, although not all nestin-positive cells expressed the ganglioside, similarly to our results *in vivo* (Figure 3).

**Figure 9:**
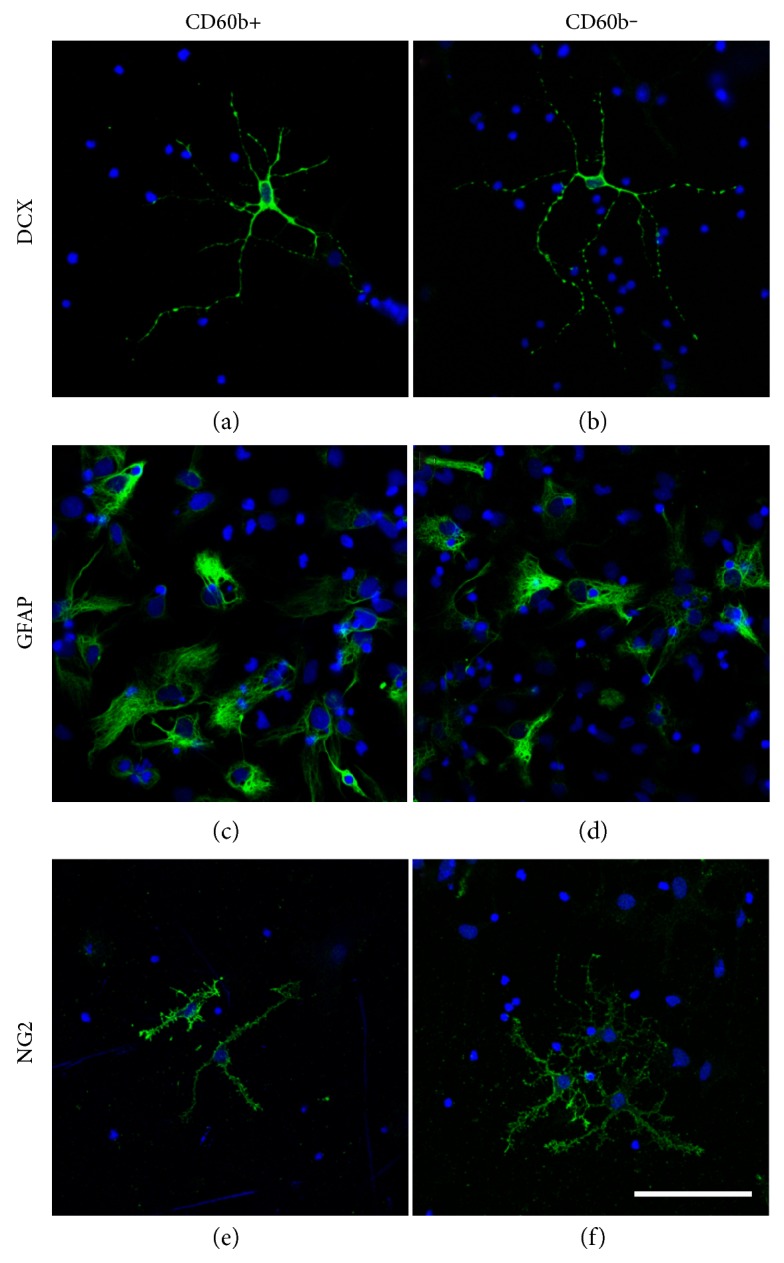
Differentiation of neurospheres derived from CD60b-positive and CD60b-negative cells. Differentiation of neurospheres originated from CD60b-positive and CD60b-negative E16 cells in DCX-positive neuroblasts (a-b), GFAP-positive astrocytes (c-d), and NG2-positive oligodendrocytes (e-f). We did not observe any clear differences between differentiated cells derived from neurospheres originated from CD60b-positive and CD60b-negative cells. Nuclei are stained with TO-PRO-3 in blue. Scale bar: 70 *μ*m.

Finally, we observed that neurospheres derived from CD60b-positive cells from the adult SVZ had the ability of self-renewal, as assessed by the formation of secondary and tertiary neurospheres (data not shown). To evaluate their multipotency, neurospheres were dissociated and plated without growth factors. After five days, we observed their capacity to generate neurons and astrocytes *in vitro* (Figure 8(d)). In this period, some cells still expressed CD60b antigens (Figure 8(b')). We also analyzed the differentiation in neuroblasts, astrocytes, and oligodendrocyte precursors comparing neurospheres derived from CD60b-positive cells and neurospheres derived from CD60b-negative cells and we did not evidence any clear difference (Figure 9).

In summary, the results indicate that from development into adulthood, the CD60b-positive cell population is enriched in neurosphere-forming neural stem/progenitor cells, compared to the CD60b-negative population.

## 4. Discussion

The ganglioside 9-O-acetyl GD3, recognized by anti-CD60b antibodies, is highly expressed during development in the rat nervous system [31], while in the adult its expression is restricted to a few regions, including the SVZ [24]. Since the SVZ persists as a neurogenic niche in the adult brain, in the present study we investigated whether CD60b antigens were expressed in neural stem/progenitor cells. We performed *in vitro* and *in vivo* analyses of CD60b expression in E14.5, E16, P0, P7, P21, and adult SVZ and found that CD60b antigens were present in neural stem/progenitor cells. We also observed that CD60b could be used to obtain an enriched population of neurosphere-forming cells.

Here, we describe the changes in CD60b expression during development and show that at E14 this ganglioside is expressed in virtually all cells in the VZ and in radial glial cell processes. Its expression decreases over time and correlates with a reduction in nestin expression. Nestin is an intermediate filament found in neural stem/progenitor cells [32], and the decrease in the expression of this protein parallels the reduction in the number of neural stem/progenitor cells in the SVZ during the transition from embryonic to adult life [7]. Interestingly, while at P0 (Figure 1), most of the cells in the dorsolateral SVZ expressed both nestin and CD60b and only a subpopulation of cells expressed both antigens at the P21 and adult stages, suggesting that CD60b is present in a subpopulation of progenitor cells. The expression of CD60b antigens in nestin-positive cells has been shown previously *in vitro* in SVZ explants from postnatal rat brains [20].

In adults, we observed the presence of CD60b in cells expressing markers of B, C, and A cells (Figure 5). Likewise, most of the antigens present in the SVZ are expressed in more than one cell type, in the neural progenitor lineage [33]. In this study, we found that CD60b was present in proliferating cells (Figures 3 and 4) but not in mature neurons (Figure 6), which agrees with the immature nature of B, C, and A cells and their shared ability to proliferate [34].

The expression of CD60b antigens was found mainly in EGFR-positive cells. EGF is one of the growth factors used to maintain neurosphere cultures. EGFR is expressed mostly in C cells, although it is also expressed in B cells [35]. Importantly, the GD3 ganglioside—the 9acGD3 precursor molecule—interacts with EGFR in membrane raft domains, protecting the receptor from degradation and contributing to the maintenance of stemness [36]. Therefore, it would be particularly interesting to test if 9acGD3, which is the main antigen recognized by anti-CD60b antibodies in the central nervous system, could also interact with EGFR.

The B cells consist of a small population with neural stem-cell properties in SVZ [8]. These cells have astrocytic characteristics, such as GFAP expression, which make it difficult to distinguish them from mature astrocytes. For this reason, although we found GFAP-positive cells expressing CD60b in the adult SVZ, we cannot affirm that they were B cells. On the other hand, if CD60b was found in astrocytes, we should have seen its expression throughout the brain, not only in the SVZ. In addition, it has been demonstrated that CD60b is not expressed in cultures of purified GFAP-positive astrocytes derived from the cerebellum, although this molecule could be found in radial glial cells [16, 37]. Our group showed previously that, in the adult, vimentin-positive cells with radial glia-like phenotype persist in the SVZ, presenting several similarities with B cells [29]. In the current work, we demonstrated that CD60b is expressed in these radial glia-like cells in the SVZ. Therefore, it is most likely that the ganglioside is indeed expressed in B cells.

Finally, we identified CD60b in A cells, which might be related to its previously characterized function in cell adhesion and migration. Negreiros and coworkers showed colocalization of CD60b antigens and *β*1 tubulin in growth cones, suggesting a role in cell adhesion [38]. Moreover, Miyakoshi and colleagues demonstrated that the immunoblockage of CD60b in postnatal SVZ explants inhibits neuroblast migration [20].

Glycosphingolipids, gangliosides in particular, are important components of lipid rafts, special membrane regions where signaling proteins, cell-adhesion molecules, and lipids are clustered [39]. It has been shown that caveolin-1, a resident lipid raft protein, is present in the embryonic VZ, caveolin-1-containing lipid rafts are involved in the coordination and coupling of *β*1-integrin, Notch1, and epidermal growth factor receptor (EGFR) signaling pathways in neurospheres [40], and phosphatidylglucoside-cointaining lipid rafts (PGLRs) serve as a platform for EGFR signaling in glial progenitors during brain development [41]. In this regard, GD3 was shown to interact with EGFR, controlling neural stem cell self-renewal [36]. Further studies are needed to elucidate the physiological role of CD60b antigens (9aGD3, in particular) in the adult SVZ niche.

Programmed cell death is an important mechanism controlling the number of neural progenitors and postmitotic neurons during brain development [42]. In this regard, the ganglioside 9acGD3 has an antiapoptotic effect on several cell lines and on lymphoblasts of patients with childhood acute lymphoblastic leukemia [43, 44]. Moreover, deacetylation of endogenous 9acGD3 induces apoptosis in a human glioblastoma cell line that expresses high levels of 9acGD3 [45]. It is possible that 9acGD3, here observed in neuronal progenitor cells, could be involved in the regulation of programmed cell death in SVZ.

The 9acGD3 function in adult SVZ neurogenesis could also be important in regulation of stem/progenitor cell adhesion and proliferation. It has been demonstrated that *α*6*β*1 integrin is expressed in adult NSC and plays a role in NSC adhesion to vasculature. The blockage of this receptor alters NSC proliferation and impairs their vascular adhesion [46]. The colocalization between 9acGD3 and *β*1 integrin has been demonstrated before in growth cones [38]. We suggest that 9acGD3 could interact with *α*6*β*1 integrin in the SVZ, probably in glycosynapses and could contribute to cell adhesion/proliferation. However, this hypothesis still has to be tested.

The presence of CD60b in neural stem/progenitor cells was supported by the neurosphere assay, which showed that CD60b-positive cells were able to form more neurospheres than were CD60b-negative cells. It is known that neurospheres are formed mostly by rapidly proliferating C cells [35]. Therefore, the ability of SVZ CD60b-positive cells form more neurospheres than do CD60b-negative cells agrees with the fact that CD60b is found mainly in cells expressing EGFR, a C cell marker.

Currently, neural stem cells do not have a specific biomarker, and therefore, a selection of prospective markers could be used as a strategy for the isolation of neural stem cells. Several research groups have found different antigens, such as LeX and *β*1 integrin that could be used to isolate or enrich neural stem cells [47, 48]. However, none of the described markers is specific for neural stem cells. Another possibility would be the use of negative markers to exclude cells that are not neural stem cells. Rietze and coworkers using this approach showed that cells expressing low levels of PNA-binding and HSA proteins could enrich significantly a population of neural stem cells [27]. In our work, we observed that CD60b-positive cells did not express PNA, corroborating with their presence in neural/progenitor cells. Pastrana and coworkers showed that neural stem cells could be purified using a combination of markers such as EGFR, CD24^low^, and GFAP, instead of using a single specific marker [33]. Nakatani and coworkers showed that GD3 ganglioside could also enrich a population of neural stem/progenitor cells and that the expression of this molecule decreases in differentiated cells [49]. Similarly, in the present study, we showed that anti-CD60b antibodies (which recognizes 9acGD3) could also be used to enrich a population of neural stem/progenitor cells, from development until adulthood.

It should be noted that, in a previous study, we showed that CD60b is highly expressed in the developing rat hippocampus and that CD60b was no longer expressed in the adult hippocampus, even after the induction of seizures with pilocarpine [17]. This observation is not completely surprising, however, considering other fundamental differences between neural stem/progenitor cells from the adult SVZ and those that are present in the adult hippocampal subgranular zone [50].

Interestingly, Yang and coworkers showed that anti-CD60b antibodies, in addition to recognizing 9acGD3, could also recognize other proteins such as *β*1 integrin receptors expressed by cerebellar neuroblasts [51]. Nevertheless, in our hands, the presence of this epitope in proteins was never detected in the developing or adult nervous system, although exhaustively investigated by SDS-polyacrylamide gels from protein extracts of different regions of the developing brain [15, 52]. Furthermore, 9acGD3 antigens were prominent in chloroform/methanol extracts of the same tissues and enzymatic treatments indicated that the epitope was sensitive to neuraminidase but not to proteases, thus indicating the glycolipid origin of all anti-9acGD3-reactive antigens [15]. Moreover, the same carbohydrate-dependent epitope recognized by anti-9acGD3 antibodies, in the nervous system, was described in T cells and hematopoietic precursors and was named CD60b. However, in these blood cells, it was described that the epitope is present in the 9acGD3 and also on proteins of 92 and 70 kDa [53, 54]. Therefore, anti-9acGD3 antibodies can recognize protein epitopes in T cells and hematopoietic precursors but there are no evidences that it does so in the developing or adult nervous system. The complete absence of staining in the SVZ of perfused (in which blood cells are absent in the brain) GD3 synthase-null mice, that exclusively lack all b- and c-series gangliosides [55], supports that hypothesis (data not shown). Moreover, we have recently shown that cerebellar progenitors cells extract from the same ganglioside null mice do not react at all with anti-9acGD3 antibodies [21].

The present study found fewer formed neurospheres than reported by other investigators. This difference might be related to the cell density used in cultures, which can vary widely among studies. It has been shown that when cell density is too high, cell fusion occurs more frequently, and chimeric neurospheres are formed [56]. Also, this study was performed in rats, whereas most studies that use the neurosphere assay have been done in mice. Ray and Gage, comparing the properties of mouse and rat neural stem/progenitor cells, found many differences in cell proliferation in response to various substrates and factors [57]. For this reason, the number of neurospheres generated in rat SVZ cultures cannot be compared to those generated in mouse SVZ cultures.

## 5. Conclusions

The possibility of modulating neurogenesis, for instance by using growth factors, or of isolating neural stem/progenitor cells that could be transplanted as a cell-therapy product has important clinical implications. Although a few cell surface markers, such as LeX and CD133 [47, 58–60], can be used for the identification and isolation of neural stem/progenitor cells, none of them is a specific marker when used alone. Here, we showed that CD60b antigens, which are abundant during CNS development and mainly related to neuronal migration, are strongly expressed in the VZ/SVZ during rat cortical development. We demonstrated how the expression of CD60b decreases over time, remaining in the adult SVZ stem cell niche, and showed that anti-CD60b antibodies could be used to isolate a cell population enriched in neural stem/progenitor cells from the developing and adult brains. Our results suggest that CD60b could be used as an additional cell surface marker to identify neural stem/progenitor cells in the embryonic and postnatal VZ/SVZ in rats. A better understanding of adult neurogenesis may not only contribute to providing new knowledge about mammalian neurophysiology, but also increase the possibility of finding new treatments aimed at regenerating CNS tissue after neurological diseases or brain injury.

## Abbreviations

9acGD3: 9-O-Acetyl GD3 ganglioside
SVZ: Subventricular zone
CNS: Central nervous system
VZ: Ventricular zone
DMEM-F12: Dulbecco's Modified Eagle's Medium/Nutrient Mixture F-12
BSA: Bovine serum albumin
PBS: Phosphate-buffered saline.
